# Assessment of macular findings by OCT angiography in patients without clinical signs of diabetic retinopathy: radiomics features for early screening of diabetic retinopathy

**DOI:** 10.1186/s12886-022-02492-x

**Published:** 2022-06-27

**Authors:** Mehrdad Afarid, Negar Mohsenipoor, Hossein Parsaei, Yalda Amirmoezzi, Mohsen Ghofrani-Jahromi, Peyman Jafari, Aliakbar Mohsenipour, Fatemeh Sanie-Jahromi

**Affiliations:** 1 Poostchi Ophthalmology Research Center, department of ophthalmology, Shiraz University of Medical Sciences, Shiraz, Iran; 2 Department of ophthalmology, Medical school, Shiraz University of Medical Sciences, Shiraz, Iran; 3 Department of Medical Physics and Engineering, Shiraz University of Medical Sciences, Shiraz, Iran; 4 Department of Biostatistics, Faculty of Medicine, Shiraz University of Medical Sciences, Shiraz, Iran; 5 Department of Statistical and Actuarial Sciences, University of Western Ontario, London Health Sciences Centre, Ontario, Canada

**Keywords:** Diabetes Mellitus (DM), Optical Coherence Tomography Angiography (OCTA), Retinal vascular analysis, Foveal avascular zone, Diabetic retinopathy

## Abstract

This cross-sectional study aimed to quantitatively analyze the optical coherence tomography angiography (OCTA) images using MATLAB-based software and evaluate the initial changes in macular vascular density and the distortion of the foveal avascular zone (FAZ), before the clinical appearance of diabetic retinopathy. For this purpose, 21 diabetic patients without any clinical features indicating DR, and 21 healthy individuals matched with patients based on their demographic characteristics were included. Macular thickness, macular vascular density, and morphological changes of FAZ were assessed using OCTA. The diagnostic ability of morphological parameters was evaluated by receiver operating curve analysis. The intraclass correlation coefficient (ICCC) index was used to check the consistency of the extracted values. There was no significant difference in age, gender, LogMAR visual acuity, spherical equivalent, and intra-ocular pressure amongst patients and controls. No correlation was found between age and the FAZ area as well as vascular density. The vascular structure of the superficial layer showed FAZ enlargement, reduced vascular density in the macular area, and significant deviations of FAZ shape parameters (convexity and Frequency Domain Irregularity) in patients compared with healthy individuals. Measurements were highly correlated between separate imaging sessions with ICCC of over 0.85 for all parameters. The represented data suggests that radiomics parameters can be applied as both an early screening tool and guidance for better follow-up of diabetic patients who have not had any sign of DR in fundoscopic exams.

## Introduction

The global incidence of diabetes is increasing rapidly. By 2030, the world's diabetic population is projected to increase to 439 million adults [1]. In diabetes, both macro-and microvascular complications occur in the course of the disease [2]. Diabetic retinopathy (DR) is the most common and potentially devastating problem among diabetics. Therefore, the diagnostic criteria for diabetes are mainly based on blood glucose thresholds that are associated with microvascular disease, especially in retinopathy [3, 4].

Approximately, 3.7 million people worldwide have a visual impairment, and 800,000 are blind from DR. This progressive disease is classified into three categories: no apparent DR, non-proliferative DR (mild, moderate, severe), and proliferative DR [5].

Many attempts were made to reduce the effect of DR [6, 7], but they were not effective. Hence, this disorder is still the main cause of visual impairment and blindness in adults [6, 7]. The main reason could be the ambiguity of DR progress over time and the lack of screening methods to identify DR in its early stages. Therefore, early diagnosis of diabetic retinopathy requires regular monitoring. Diabetic retinopathy screening intervals vary depending on the duration of diabetes, age, and type of diabetic retinopathy. Vision-threatening diabetic retinopathy treatments minimize the risk of blindness if applied in the early stages. This fact underscores the importance of screening methods for diagnosing treatable stages. Current screening is generally based on clinical findings in dilated ophthalmoscopy [8, 9]. The integration of vascular changes obtained by optical coherent tomography (OCTA) angiography with current screening methods seems to assist the diagnosis process. Several studies have used vascular parameters derived from OCTA to evaluate the efficacy of retinopathy treatment [10].

OCTA is a new non-invasive diagnostic technique for visualizing the retinal vasculature in the macular region. Recent studies have shown that OCTA may be useful in monitoring disease progression in DR [11] and revealed some abnormalities in the Foveal Avascular Zone (FAZ) area, and vascular structure [12, 13]. Nevertheless, previous studies did not present reliable parameters to be used in the DR screening test. This project aimed to study the effectiveness of OCTA in detecting early pathological changes in the retina in diabetic patients by using MATLAB-based software and measuring several radiomics indices such as macular vascular density and parameters related to FAZ shape.

## Methods

### Ethics

The presented cross-sectional study was carried out at Shiraz University of Medical Sciences (SUMS). The protocol used was consistent with the directive of the Helsinki Declaration and approved by the Ethics Committee of Shiraz University of Medical Sciences (Ethics number: IR.SUMS.MED.REC.1397.036) and informed consent was obtained from the participants in the study. Macular vascular findings were evaluated with an OCT Angiogram (Heidelberg Engineering SPECTRALIS OCTA). We measured several quantitative variables of vascular density and FAZ; including area, form factor, roundness, extent, solidity, convexity, and irregularity. These parameters were measured using a developed MATLAB-based software.

### Subjects

Image findings were selected from 41 eyes of twenty-one patients, (11 women and ten men) with type 2 DM without DR and 41 eyes of twenty-one healthy individuals (11 women and ten men). Matching was conducted based on age and gender.

The inclusion criteria were as follows: 1) Confirmed diagnosis of diabetes type 2; 2) age >35 years; 3) no clinical evidence of DR (DR staging was defined according to the Clinical Diabetic Retinopathy Scale proposed by the Diabetic Retinopathy Project Group) [5]

Major exclusion criteria were as follows: 1) systemic diseases affecting the retina: Hypertension, thyroid diseases, neoplastic diseases, sickle cell disease, rheumatoid disorder,

2) Eye diseases: excessive myopia (>4), retinal vasculitis, history of cataract surgery, vitrectomy, refractive surgery, intraocular surgeries, retinal vascular accident, age-related macular degeneration (AMD), glaucoma, 3) Medications that affected the retina: chemotherapy, radiation, tamoxifen, hydroxychloroquine, chloroquine, and any other medication that might cause retinal changes.

Two eyes (one from patients and one from controls) were excluded due to the poor quality of the images. The procedure was approved by SUMS.

### Study protocol

Thorough and complete history and ophthalmological examination were performed by an ophthalmologist. Visual acuity (LogMAR visual acuity and Snellen visual acuity ratios), autorefractometry, Slit-lamp biomicroscopy, and intraocular pressure were measured.

### Image acquisition

OCTA and OCT (standard OCT with thickness maps) scans were acquired using a commercially available Heidelberg Engineering SPECTRALIS OCTA Module device following standard instructions for Image acquisition. All images were En face type, with a field of view of 10°x10° (~ 2.9 x 2.9 mm) in high-resolution mode (~5.7 μm/pixel). This scan pattern (512 A-scans x 512 B-scans) provides the resolution needed to visualize the smallest capillaries by providing a more distinctive assessment of vascular abnormalities at the capillary level in the superficial and deep vascular complex. The superficial vascular complex consists of nerve fiber layer vascular plexus and superficial vascular plexus. The deep vascular complex encompasses intermediate and deep capillary plexus [14].

### Quantitative analysis

The collected OCTA images were analyzed quantitatively, using an interactive MATLAB-based software developed by the authors. The detailed description of the SOFTWARE and the OCTA parameters along with the definition and equations used in this work are provided in Reference no [15] available from (https://jbpe.sums.ac.ir/article_48006.html). To prevent reparation, here, we did not repeat defining these parameters, instead, we focused on the applications of these indices and software. Particularly, several indices describing microvascular morphology, vessel morphology, and FAZ morphology were measured. For FAZ morphology analyses, we measured several parameters related to both size and shape of FAZ; including area, perimeter Feret’s diameter circularity, axial ratio, roundness, and solidity [16, 17].

### Vascular density

The retinal vascular density was calculated in two circular regions: the foveal region and the parafoveal region. Vascular density was calculated in four regions superior nasal, inferior nasal, inferior temporal, and superior temporal. The FAZ region was omitted when calculating vascular density to enhance diagnostic accuracy.

### Macular thickness

Based on the Early Treatment Diabetic Retinopathy Study (ETDRS) map macular thickness for five regions (circle diameters: 1mm, 3 mm, and the center of foveal) was documented for each subject [18, 19].

### Test-retest of OCTA device

Assessment of reproducibility is indispensable for the evaluation of any imaging modality, which is the operator and/or subject dependent. Therefore, we assessed 16 eyes (5 controls and 3 cases) to calculate reproducibility. Images were taken in separate sessions.

### Statistical analysis

The Shapiro-Wilk test was used to check normative distribution. Data were quantitative and presented as mean ± SD. The test-retest of the OCT Angiography device and the correlation between left and right measures of the same patient eyes are modeled, using random-effect models. An independent-samples t-test was used to compare demographic features. The Pearson correlation coefficient was applied to analyze the correlation between the FAZ area and demographic features. ROC curve analysis was applied to determine cutoff values for irregularity and convexity. Bonferroni correction was performed to assess multiple comparisons between groups and results. P-value < 0.004 was considered to be statistically significant. Analyses were done, using SPSS, Version 25.

## Results

The demographic characteristics of the individuals who participated in this study are summarized in Table 1. In the present study, the OCTA findings of diabetic patients -who had no sign of retinopathy- were compared with the control group without diabetes. Although the duration of diabetes or the way of diabetes management might affect the retinopathy, these were not the purpose of our study. This study focused on the question of whether a patient with diabetes (regardless of how diabetes is controlled or the duration of diabetes) who has no clinical evidence of retinopathy shows any microvascular changes in the macula compared to controls.

**Table 1:** Demographic characteristics in healthy individuals and patients including age, sex, logMAR visual acuity, spherical equivalent, and IOP are compared

Parameters	Controls (n:21)	Patients (n:21)	*P* value
**Age (years)**	53.71 ± 7.06	54.62 ± 7.20	0.683^a^
**Sex (F: M)**	11:10	11:10	
**Duration DM (years)**	N/A^b^	8.76 ± 6.03	
**LogMAR visual acuity**	0.00 ± 0.037 (10/10)	0.00 ± 0.076 (10/10)	0.797
**Spherical equivalent**	-0.18 ± 0.98	-0.03 ± 1.18	0.534
**IOP (mmHg)**	13.88 ± 3.16	13.50 ± 1.78	0.499

^a^Independent-sample t-test

^b^Not applicable

There was no correlation between age and FAZ area in superficial and deep layers. (*r*=0.038, *P*=0.735 and *r*= 0.114, *P*=0.305, respectively). There was also no correlation between age and vascular density in both layers. (*r*=-0.054, *P*=0.629 in superficial layer and *r*=-0.052, *P*=0.639 in deep layer).

### Macular thickness

Macular thickness was thinnest at the center of the fovea in both groups. The macular thickness of the assessed regions showed no significant difference between patients and controls (*P*>0.004) Table 2.

**Table 2:** Macular thickness based on ETDRS protocol in the center of the fovea, 1 mm and 3 mm region

Macular region	Control (41eyes) (Mean ± SD)	Case (41eyes) (Mean ± SD)	*P*-value
**Center of fovea**	224.60 ± 22.94 μm	216.68 ± 18.73 μm	0.05
**1 mm ETDRS**			
**Fovea**	264.56 ± 19.59 μm	258.12 ± 24.07 μm	0.09
**3 mm ETDRS**			
**Temporal**	327.36 ± 10.92 μm	319.78 ± 13.83 μm	0.01
**Nasal**	339.43 ± 17.97 μm	331.12 ± 16.67 μm	0.04
**Superior**	341.80 ± 11.99 μm	331.17 ± 16.51 μm	0.013
**Inferior**	334.56 ± 14.15 μm	326.60 ± 16.62 μm	0.046

### Macular vascular density

Macular vascular density assessment showed a significant reduction in superficial vascular density in ~ 2.9 x 2.9 mm square area in DM patients (44% ± 6% vs. 52% ± 6%, *P*<0.001). Vascular density in the deep layer did not experience any significant reduction (42% ± 5 vs. 47% ± 3%, *P*> 0.004).

The foveal and parafoveal areas were evaluated in four quadrants. Foveal vascular density was reduced significantly in the inferior temporal region of the superficial layer (*P*=0.004). There was no significant reduction in the deep layer of the foveal zone Table 3. There was a significant reduction in the vascular density of the parafoveal zone in all four regions of the superficial layer. No significant reduction was observed in the vascular density of the parafoveal zone of the deep layer Table 4.

**Table 3:** Foveal vascular density is defined as a circle with a 1mm diameter from the center of the macula

Inner ring (1 mm diameter)		Control (41eyes) (MEAN ± SD)	Case (41eyes) (MEAN ± SD)	*P*-value
**Superior** **nasal**	Deep	20% ± 8%	20% ± 11%	0.03
	Superficial	38% ± 12%	32% ± 13%	0.01
**Superior** **temporal**	Deep	19% ± 7%	16% ± 7%	0.04
	Superficial	38% ± 12%	30% ± 13%	0.005
**Inferior** **temporal**	Deep	19% ± 7%	16% ± 8%	0.01
	Superficial	39% ± 12%	30% ± 16%	0.002
**Inferior** **nasal**	Deep	19% ± 8%	16% ± 9%	0.05
	Superficial	37% ± 11%	29% ± 15%	0.02

**Table 4:** Parafoveal vascular density is defined as a circle with a 2mm diameter from the center of the macula

Outer ring (2 mm diameter)		Control (41eyes) (MEAN ± SD)	Case (41eyes) (MEAN ± SD)	*P*-value
**Superior** **nasal**	Deep	50% ± 6%	46% ± 10%	0.02
	Superficial	48% ± 8%	41% ± 8%	<0.001
**Superior** **temporal**	Deep	49% ± 5%	43% ± 11%	0.009
	Superficial	48% ± 8%	41% ± 8%	<0.001
**Inferior** **temporal**	Deep	46% ± 5%	41% ± 9%	0.01
	Superficial	51% ± 8%	40% ± 8%	<0.001
**Inferior** **nasal**	Deep	47% ± 5%	41% ± 9%	0.01
	Superficial	51% ± 8%	39% ± 9%	<0.001

### FAZ area

The mean FAZ area was 0.357 ± 0.092 mm^2^ in the control group and 0.442 ± 0.147 mm^2^ in the patient group for the superficial layer and the deep layer was 0.334 ± 0.1 mm^2^ in the control group and 0.391 ± 0.12 mm^2^ in patients. The FAZ area was significantly larger in diabetic patients in the superficial layer (*P*<0.001). The FAZ area in the deep layer was not significantly enlarged (*P*=0.02).

### Descriptive factors of FAZ shape

Amongst the quantitative factors of the FAZ shape, there was a significant difference between diabetic patients and healthy subjects in the frequency domain irregularity and convexity within the superficial vascular complex layer (*P*<0.004). The other factors did not show a marked difference between patients and controls Table 5.

**Table 5:** Parameters of the FAZ shape

Parameters		Control (41eyes) (MEAN ± SD)	Case (41eyes) (MEAN ± SD)	*P* -value
**Form** **factor**	Superficial	0.32 ± 0.06	0.29 ± 0.07	0.009
	Deep	0.62 ± 0.11	0.67 ± 0.08	0.023
**Roundness**	Superficial	0.85 ± 0.08	0.84 ±0.06	0.286
	Deep	0.82 ± 0.09	0.82 ± 0.08	0.933
**Extent**	Superficial	0.63 ± 0.04	0.63 ± 0.03	0.700
	Deep	0.65 ± 0.08	0.67 ± 0.04	0.171
**Convexity**	Superficial	0.62 ± 0.05	0.59 ± 0.06	0.004
	Deep	0.85 ± 0.05	0.88 ± 0.03	0.029
**LS** **Ratio**	Superficial	1.14 ± 0.10	1.2 ± 0.08	0.330
	Deep	1.18 ± 0.11	1.19 ± 0.10	0.666
**Solidity**	Superficial	0.86 ± 0.03	0.86 ± 0.03	0.481
	Deep	0.90 ± 0.04	0.91 ± 0.03	0.133
**Irregularity**	Superficial	0.12 ± 0.04	0.13 ± 0.02	0.149
	Deep	0.13 ± 0.05	0.11 ± 0.03	0.039
**Frequency** **domain irregularity**	Superficial	152.28 ± 80.55	208.63 ± 84.98	0.001
**Irregularity**	Superficial	0.12 ± 0.04	0.13 ±0.02	0.149

### Diagnostic ability of FAZ shape parameters

#### Irregularity

**Fig. 1:**
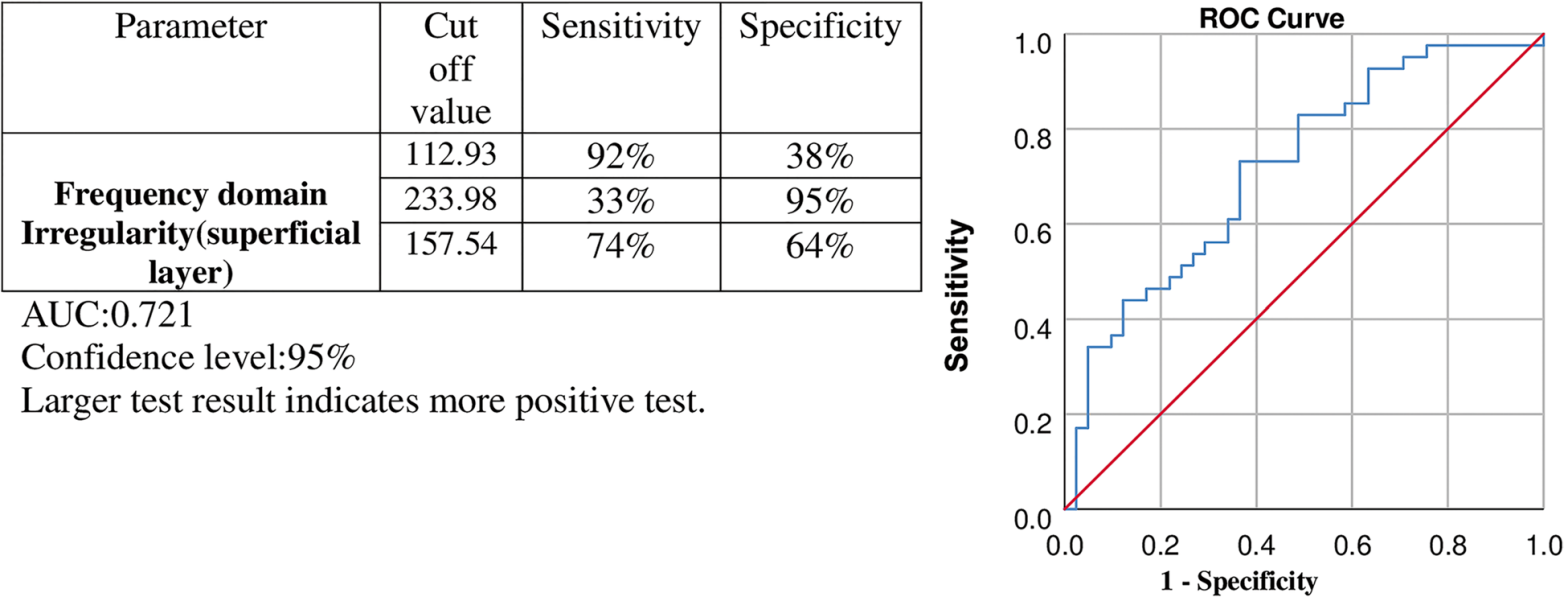
Evaluation of the result for the diagnostic ability of frequency domain irregularity of FAZ to differentiate diabetic eyes from healthy ones

Irregularity in the superficial region was evaluated as frequency domain irregularity, which is defined as the dispersion of each FAZ border point from the center (refer to Reference no [15] for more details). Frequency domain irregularity with a cutoff value above 157.54 showed 73% sensitivity and 63% specificity in the superficial layer for differentiation of a diabetic eye from a healthy one (area under the curve (AUC) of 0.717) (Fig. 1).

#### Convexity

**Fig. 2:**
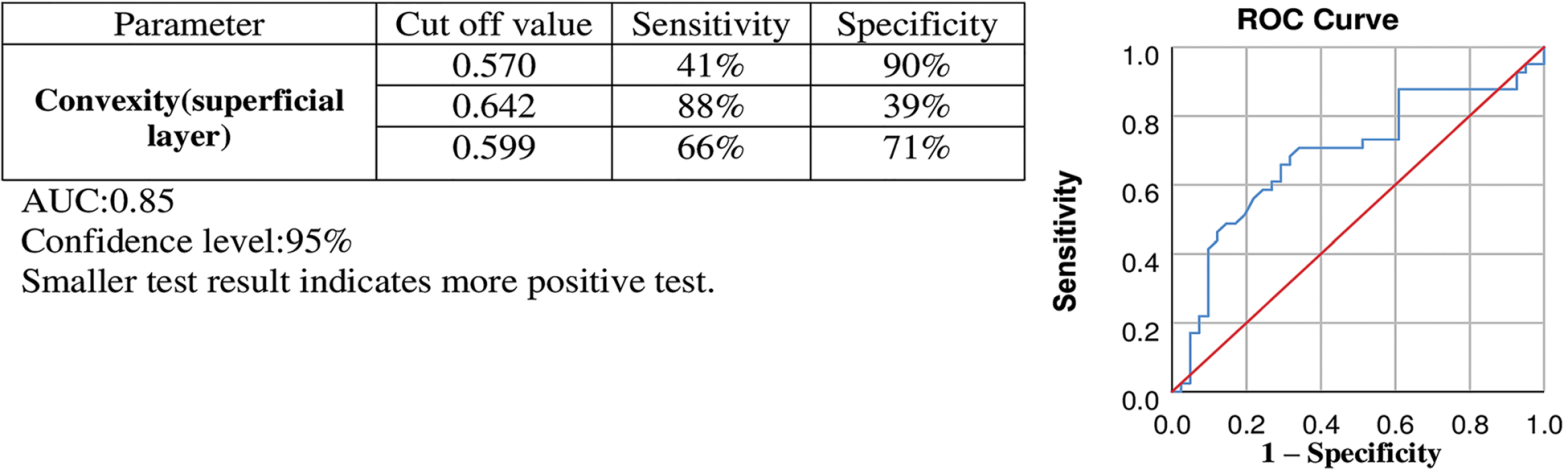
Evaluation of result for the diagnostic ability of convexity of FAZ for differentiating diabetic eyes from healthy ones

Convexity illustrated dimensionless quantities of FAZ, independent of its size (refer to Reference no [15] for more details). Convexity with a cutoff value below 0.599 showed 66% sensitivity and 71% specificity in the superficial layer for differentiation of a diabetic eye from a healthy one. (AUC of 0.685). Other cutoff values with higher sensitivity or specificity are shown in Fig. 2.

### Test-retest of OCTA device

Vascular density, FAZ size, and foveal thickness were highly correlated for the same subject when measured through separate imaging sessions, with intraclass correlation coefficients of over 0.85 for all assessments, reflecting excellent reproducibility. The ICC of each parameter is summarized in Table 6.

**Table 6:** Reproducibility of OCTA measurements in 16 eyes

	ICC^a^	95% CI^b^
**Superficial** **vascular** **density**	0.964	0.901-0.987
**Deep** **vascular** **density**	0.888	0.709-0.959
**Superficial** **FAZ** **area**	0.976	0.933-0.992
**Deep** **FAZ** **area**	0.983	0.952-0.994
**Foveal** **thickness**	0.928	0.892-0.987

^a^Intraclass correlation coefficient

^b^Confidence interval

## Discussion

DR is one of the most common and destructive causes of visual acuity around the world, So the early diagnosis of this disorder may assist with its treatment and management. Research shows that in the first few days to the first few weeks of diabetes, pathological changes occur in the retina that eventually leads to microvascular complications. This fact shows that even low blood sugar levels in pre-diabetes are harmful to the retinal vasculature [20]. Therefore, early detection of DR in the early stages can assist in the diagnostic thresholds, timely treatment, and follow-up intervals. Calculating morphological parameters is a noninvasive technique for quantitative assessment of macular perfusion status with potential clinical application in diabetic patients. Blood sugar and HbA1c are currently used to diagnose diabetes, however, several factors can affect these measurements. HbA1c can be misleading in various medical conditions (e.g. hemoglobinopathies, iron deficiency, hemolytic anemia, severe hepatic or renal diseases) or FBG and 2hPG in a 75 g OGTT have high day-to-day variability [21]. Since macular involvement and macular edema are very important and common causes of vision loss in diabetic patients at any stage of diabetic retinopathy, we conclude that OCTA may be an important and effective tool for early detection of macular involvement. On the other hand, in clinical evaluation, sometimes minor changes are not visible, and with early detection of small changes in the macula, the periods and timing of subsequent follow-up or patient recommendations might be changed. We hope these structural changes can be used to determine diabetes at an early stage in conjunction with other measures. However, the most significant aspect of these parameters is the detection of vascular and anatomical changes in the retina so that further damage can be prevented. As a result of these changes, we hope that procedures can be applied effectively and efficiently to prevent further damage. The present study focused on the primary vascular changes in diabetic patients with no manifestation of DR in clinical examination by analyzing the OCT images from normal and diabetic patients using MATLAB-based software. Previous studies have discovered considerable variations in macular thickness amongst subjects of different races, genders, and ages. These variations depend on using different types of OCT (time-domain vs. spectral domain) [19, 22, 23]. Our study showed no significant decrease in the macular thickness in any region. However, previous studies have shown that retinal nerve cell injury begins even before the clinical manifestations of vascular complications [24]. To interpret our result we considered two conditions, first because no change was found in the macular thickness it could be due to the well-controlled glycemic status of patients another possibility is gradual microscopic vascular changes can also lead to increased permeability of the vessels, and subsequently concealed edema can mask retinal nerve fiber layer (RNFL) loss. Reduced macular thickness is indicative of diabetes-related neuropathy onset and RNFL loss in the early stages of diabetic retinopathy. Several previous studies have emphasized the relationship between the RNFL thickness and serum neurotrophic factors level in the early stage of retinal pathologies [25–28].

Our analysis revealed a significant reduction in macular vascular density in the superficial layer. This finding is in line with previous studies that showed vascular density decreases in the early stages of diabetic retinopathy [11–13, 29–31]. The vessels of the parafoveal region indicate the border of the FAZ region, and the ischemic changes following retinopathy in this area can be seen as reduced vascular density or changes in FAZ shape and size. There was a significant vascular density reduction in the inferior temporal region of the superficial parafoveal zone, showing that this area is more susceptible to ischemia. However, proof of this claim requires broader studies with a larger sample size. To explain this observation and correlate it with the reports on vascular endothelial growth factor (VEGF), it is noteworthy to say that when diabetic retinopathy develops, first the walls of the arteries and then the endothelial cells are damaged. This leads to blockage of the retinal capillaries and subsequent ischemia. Many people with diabetes experience this condition, called non-proliferative DR. Therefore, if we photograph the patient during the non-proliferative DR stage, we expect the retinal vascular density to be low. After this stage, due to ischemia, the VEGF factor is secreted, which causes angiogenesis. VEGF plays an important role in causing retinal vasculopathies. Under ischemic conditions and due to the decreased BDNF levels, neurons and retinal vascular cells are destroyed and proinflammatory cytokines are released into the environment [32, 33]. These microenvironmental changes in the retina increase VEGF expression [34]. The process of angiogenesis stimulates the production of blood vessels to recruit the circulating neurotrophins in the blood to the site of injury and regenerate damaged cells. Thus, increased retinal VEGF level in DR patients occurs in response to inflammatory cytokines and facilitate retinal neural regeneration [35]. However, this angiogenesis does not occur everywhere and usually occurs locally in some parts of the retina; While in other retinal regions, the vascular density is still low. Therefore, we expect vascular density to be generally lower in diabetic patients than in non-diabetic individuals. Based on the available evidence, as well as our data in this study, which reported a change in vascular morphology in the early stages of DR, it seems that the damage to retinal vascular cells due to the metabolic condition leads to morphological vascular changes in the macular region which confirms our observation.

Previous measurements indicated that the FAZ area undergoes a gradual size increase as DR progress [12, 13, 29, 30]. Likewise, our study, revealed that FAZ size increased in diabetic patients, besides several descriptive parameters of FAZ shape were altered in the early stages of DR in the superficial layer. In diabetic patients without DR, it is important to monitor the size of the FAZ, as increasing the size of the FAZ at the onset of the disease is a reversible step if blood sugar levels are controlled [36]. There are only a few reports about early changes in FAZ shape, using OCTA [13, 31, 37]. Lupidi et al. assessed diabetic retinopathy progression in eyes with diabetic maculopathy by presence or absence of vascular abnormality parameters and depicted perifoveal arcade disruption, linear vascular dilations, intraretinal microvascular, flow-void areas appeared much more than microaneurysms, the first clinical sign of DR [31]. In our study, various parameters were evaluated. Quantitative morphological features of the FAZ region, such as irregularity and convexity in superficial layers can be the initial point to introduce these indices as radiomic features, but more studies with larger samples in different populations are required. Our study showed that with the help of radiomics, OCTA images can be used as a screening tool to find susceptibility to DR. Radiomic features can detect the early vascular changes in DR, which are not recognizable neither by clinical examinations nor the serum level of pro-inflammatory factors. Based on these findings and clinical examination, effective preventive medical intervention can be considered [38].

Our analysis showed two significant morphological parameters of FAZ as indices of early microvascular changes, namely irregularity and convexity.

### Irregularity

Differences in FAZ irregularity between diabetic patients and healthy controls can be due to blood rheology and/or the emersion of the Pro-thrombotic state, which underlie the initiation of DR. It might also happen due to morphological responses of the vascular compartment to tissue hypoxia that ultimately leads to a significant reduction of flow in the vessels surrounding the FAZ. Quantitative translation of these changes can indicate the early stages of abnormal autoregulation of retinal blood flow [39]. This abnormal auto-regulation might be detected by frequency domain irregularity.

### Convexity

Those shapes which are more convex have more protrusions on their boundary. Convexity of the FAZ area in diabetes showed a significant reduction compared to healthy controls. Less convexity can be interpreted as fewer curved outwards with more intrusion in the FAZ area.

This change could be used as an early marker of ischemia and applied in diabetic patients as radioramic features for early diagnosis of DR.

Limitations should always be considered. The primary issue is artifacts in vascular structure in the OCTA images. However, we tried to minimize this error by analyzing each superficial and deep layer separately. Overall, applying these vascular changes as quantitative measurements by expanding them as radiomics features to define unique screen patterns for DR, necessitate further studies with a larger sample size to evaluate their practical use.

## Conclusion

Our study focuses on the early changes in FAZ shape. These parameters can detect the initiation of DR, but they can also be used as a diagnostic tool for diabetes based on macular vasculature changes. The diagnostic ability of irregularity and convexity of FAZ showed acceptable sensitivity and specificity. To the best of our knowledge, there is no report on applying these changes as radiomics features to distinguish between diabetic eyes from healthy ones. The promising view is the potential of these radiomics features for diagnosis of diabetes in a seemingly healthy person without any invasive blood sampling (provided all excluding criteria are rejected) or for the screening and surveillance of DR. By using these radiomics features, intervals of follow up of diabetic patients without obvious clinical evidence of diabetic retinopathy may also change if any abnormality is detected in each of the radiomics parameters.

The design of this study was not to evaluate the effect of age and sex on the OCTA, moreover, the age range in the control and case groups was limited. To better assess the effect of age and gender, a larger sample size with different age groups might be required.

## Data Availability

The datasets used and/or analyzed during the current study available from the corresponding author on reasonable request
